# Letter to the Editor: "Evaluate the effects of curcumin on the prevention of atrial and ventricular arrhythmias and heart failure in patients with unstable angina"

**Published:** 2020

**Authors:** Masoud khorshidi, Sanaz Jamshidi, Mohammadreza Vafa

**Affiliations:** 1 *Student Research Committee, Department of Nutrition, School of Public Health, Iran University of Medical Sciences, Tehran, Iran*; 2 *Department of Nutrition, School of Public Health, Iran University of Medical Sciences, Tehran, Iran*

**Dear editor**

In a recent issue of the journal, Dastani et al. (2019) ▶ evaluate the effects of curcumin on the prevention of atrial and ventricular arrhythmias and heart failure in patients with unstable angina. Despite the fact that the topic was interesting, there were some defects and we wish to call to your attention important factual issues in this publication.

The most critical flaw of the article was the duration of supplement therapy which was about 5 days and the related complications such as heart failure, myocardial infarction, the rate of cardiopulmonary resuscitation, and the mortality rate were measured after this duration which we know is too short and it cannot show the complications and supplemental effect properly. It is important to know that heart failure and related complications are mostly chronic conditions and it is not accurate to claim that it is measurable in such a short time. Furthermore researchers didn’t assess blood pressure, atrial fibrillation, valvular heart disease, alcohol use and smoking in patients which can all change the structure and function of heart and can relate to heart failure (McMurray and Pfeffer, 2005 ▶).

In the title and keywords part, it is important to point out that which form of curcumin exactly was used in the study nevertheless the author’s only mention the “nanocurcumin” type at the end of abstract. 

The authors claimed that there isn’t any similar clinical study. Even if there is not any related study, the priority is working on the effect of curcumin on some basic features like lipid profile and hypertension, and then some advanced issues like angina pectoris and myocardial infarction. However we found different researches concentrated on the similar content. One of our significant questions involves the determination of sample size that could be possible by considering previous studies which wasn’t mentioned by the authors ( Rahimi et al., 2016 ▶; Rahmani et al., 2016 ▶). 

In method section, Figure 1 shows study participation diagram which wasn’t provided in the correct format. We addressed some studies with accurate participation diagram (Bauer et al., 2015 ▶; Trabal et al., 2015 ▶).

To be specific, the authors mentioned the absorption rate of SinaCurcumin in mice which is 50 times more than conventional powder of curcumin and they referenced two human studies. However there was a failure in reporting the oral absorption of SinaCurcumin in human. Furthermore, the studies that have been referenced didn’t compare the absorption rate of curcumin and nanocurcumin which there is not any justification for prescribing this dose (80 mg) in the study so it may not show proper effect.

Furthermore, duration of the intervention in this study was determined without reason and logic and the authors didn’t mention any references.

## Conflict of interest

The authors have declared that there is no conflict of interest.
